# Crosscultural Validation of the Community Integration Questionnaire–Revised in an Italian Population

**DOI:** 10.1155/2020/8916541

**Published:** 2020-08-28

**Authors:** Melissa Ioncoli, Anna Berardi, Marco Tofani, Francescaroberta Panuccio, Annamaria Servadio, Donatella Valente, Giovanni Galeoto

**Affiliations:** ^1^Sapienza University of Rome, Italy; ^2^Department of Human Neurosciences, Sapienza University of Rome, Rome, Italy; ^3^Neurorehabilitation Unit, Department of Neurosciences and Neurorehabilitation, Bambino Gesù Children's Hospital, Italy; ^4^Department of Health professions, Tor Vergata Hospital of Rome, Roma, Italy; ^5^IRCSS Neuromed, Via Atinense, 18, 86077 Pozzilli, Italy

## Abstract

**Objective:**

The aims of this study are the translation, cultural adaptation, and validation of the Community Integration Questionnaire–Revised (CIQ-R) in Italian in a group of individuals with no clinical evidence of disability.

**Methods:**

The test's internal consistency and validity were assessed by following international guidelines. The test's internal consistency was examined using Cronbach's alpha (*α*) coefficient. Pearson's correlation coefficient was calculated to assess the test's concurrent validity compared with the Short Form-12 (SF-12) health survey.

**Results:**

The CIQ-R was administrated to 400 people with no clinical evidence of disease, impairment, or disability, aged between 18 and 64. Cronbach's *α* reported a value of 0.82 in the home integration subscale. The test also showed a good test-retest reliability, with an Intraclass Correlation Coefficient of 0.78, and a significant correlation between the total score of the CIQ-R and the Physical Component Summary (PCS) of the SF-12 (*r* = 0.118), between the “social integration” subscale's score and PCS12 (*r* = 0.121) and between the “Electronic Social Networking integration” subscale's score and PCS12 (*r* = 0.184), with *p* < 0.05.

**Conclusion:**

This is the first study to report the results of the translation and validation of the CIQ-R in Italian. The CIQ-R is an important tool for Italian professionals and can be useful in both clinical practice and research for measuring the level of community integration among the healthy population.

## 1. Introduction

Redeveloping the community integration and social participation of people with disability is a key goal for all areas of rehabilitation and an important focus for all types of healthcare professionals. After an acute event, such as traumatic brain injury (TBI) or spinal cord injury (SCI), individuals experience not only a change in their functional and occupational performance but also a reduction of social integration and participation in the domestic or family environment and leisure, work, and/or school activities [1–4]. This is due not only to the symptoms related to the disease but also to a number of factors, such as the presence of secondary issues related to cognitive, mental, and mobility aspects; demographic variables (e.g., age, sex, race, and culture); level of education, economic situation, social support, and environmental barriers that alter the usual occupational roles and negatively influence individuals' Quality of Life (QoL) [2, 5].

Therefore, the definition of “community integration” also includes basic elements, such as interpersonal relationships, independence, and the possibility to participate actively in meaningful daily life activities [6]. The importance of community integration's features, besides its deep correlation with QoL [7, 8], implies that an accurate measurement of the construct is imperative for rehabilitation. This means that it would be necessary to consider an environmental assessment as a specific intervention to guarantee the patient's social reintegration and participation in meaningful occupations.

Several scales have been developed and used to assess the patient's degree of community integration, such as the Craig Handicap Assessment and Reporting Technique (CHART) [9], the Community Integration Measure (CIM) [10], the Sydney Psychosocial Reintegration Scale (SPRS) [11], and the Community Integration Questionnaire–Revised (CIQ-R) [2]. Nowadays, the CIQ-R is the most commonly used outcome tool for measuring the participation and community integration of people with disability, especially in the population with TBI. The authors chose the CIQ-R for the following reasons: (1) it allows us to obtain an objective overview of community integration and participation in the activities of daily living (ADL) and instrumental activities of daily living (IALD), (2) it is brief to administer (takes 10–15 minutes), (3) it can be administered directly to the person—either face-to-face or over the telephone—or self-administered, but it can also be completed by proxy. Moreover, it is weighted towards measuring behaviours as opposed to feelings, sensitive to different conditions and daily life activities, and easy for healthcare professionals to use.

In his studies, Willer was the first to evidence the necessity of a measurement of community integration in the rehabilitation of neurological trauma, defining community integration as “effective role performance in community settings” [5]. In 1994, he and his colleagues developed the Community Integration Questionnaire (CIQ). In 2014, an Australian group of researchers worked with Willer to revise the CIQ to include three new items for evaluating electronic social networking (ESN), update the scoring, and publish the CIQ-R and large-scale normative data concerning adults of working age [2].

In the last few years, the CIQ-R has been used in many researches worldwide [12–20] and translated and validated in several languages, such as Persian [21], Japanese [22], and Croatian [23]. Despite the CIQ-R's wide use and the many articles comparing the CIQ with other evaluation scales (e.g., the CHART [24], SPRS [25], CIM [26], and Functional Independence Measure (FIM) [27]), studies attesting the reliability and construct validity of the CIQ, compared to those of different tools and gold standards, are limited. Moreover, in terms of the Italian population or context, there are currently limited validated scales available for measuring community integration. The first version of the CIQ has been validated in Italy [25]; however, as evidenced by the literature, an Italian version of the CIQ-R is not yet available, so the authors decided to conduct the study of validation in the Italian language. In recent years, the interest in the translation and cultural adaptation of outcome measures has grown. [28–35] The aims of this study are the translation, cultural adaptation, and validation of the CIQ-R in Italian in a group of individuals with no clinical evidence of disability.

## 2. Methods

This study was conducted by a research group composed of medical doctors and rehabilitation professionals from the “Sapienza” University of Rome and the “Rehabilitation & Outcome Measure Assessment” (R.O.M.A.) association. In the last few years, the R.O.M.A. [36–42] association has dealt with the validation of many outcome measures in Italy.

This study was divided into two stages. First, the original English version of the CIQ-R was translated into Italian [43]. Second, the translated CIQ-R was tested for validity and reliability in a prospective study following international guidelines [44].

### 2.1. Instruments

The CIQ-R is an 18-item questionnaire designed to be a brief, reliable measure of home, social, productivity integration, and electronic social networking, designed at first to be administered to people with TBI [12–14, 45–47]. In recent times, it has also been used to assess people with SCI [3, 15, 24, 48] and other physical disability [16], aphasia [8, 49], brain tumours [17], and burn injuries [50], as well as young people with a range of acquired and congenital disabilities who are living in nursing homes for the elderly [51].

The instrument is divided into four subscales that measure, respectively:

1. *Home integration*: active participation of the person in the activities of the home.
2. *Social integration*: participation in a variety of activities outside the home and interpersonal relations.
3. *Productivity*: involvement in employment, education, and volunteer activities.
4. *Electronic Social Networking (ESN)*: participation in electronic social networking.

Scores are primarily created by self-report of performance frequency, with additional weight given on whether assistance was obtained. Most CIQ-R items are scored from 0 to 2, and the subtotals for home integration (0–12), social integration (0–10), productivity (0–7), and ESN (0–6) subscales are calculated, as well as the total score (0–35). A higher score indicates a higher level of community integration.

The CIQ-R can be completed directly with the person or by proxy. It takes approximately 10-15 minutes to complete. It can be administered in person, by telephone, or self-administered.

The Short-Form 12 (SF-12) health survey, used as the gold standard, is a brief, self-administered, generic measure of health-related quality of life developed to be a shorter, yet valid, alternative to the original form, the SF-36. The 12 items in the SF-12 allows to get a Physical Component Score (PCS) and a Mental Component Score (MCS), in which a higher score indicates a better health status [52].

### 2.2. Translation Process

Once the consent of the developers of the original version of the CIQ-R [2] was received, the original tool was translated from English to Italian, following the “Translation and Cultural Adaptation of Patient Reported Outcomes Measures–Principles of Good Practice” guidelines [43]. The translation process included three steps. Firstly, two native English official translators have independently translated the questionnaire into Italian (forward translation). Subsequently, one native Italian translator chose the best translation in order to create the final Italian version of the CIQ-R. Then, a bilingual person with a certificated knowledge of the English language translated the text back into English in order to have the approval of the Italian version by the author.

### 2.3. Participants

Individuals recruited from various Italian universities, hospitals, and other community places were asked to involve the study. The inclusion criteria adopted were the same as defined by the original validation study [2], in which participants have to (1) have an age between 18 and 64, (2) have no clinical evidence of disability, and (3) have the ability to understand Italian instructions in order to correctly complete the questionnaire. Individuals hospitalized, or with serious and disabling pathologies, were excluded in order to record the first normative values of the instrument on healthy people.

Before administering the test, each subject was briefed and signed a written consent for releasing their personal data about gender, age, level of education, annual household income, area of residence, and living situation.

### 2.4. Validation Procedures

Participants were asked to complete the CIQ-R first, and then the SF-12, used as the gold standard for health-related quality of life assessment. After the first administration of both tests, a subgroup was tested twice. All questionnaires were self-administrated. In order to assess test-retest reliability, the time interval needs to be sufficiently long to prevent recall; the interval chosen between the repeated administration processes was 14 days. All statistical analyses were performed using the Statistical Package for the Social Sciences (SPSS) version 23.0 for Windows, by the International Business Machine Corporation (IBM). The descriptions of the variables were carried out by using frequency tables, means, and standard deviation (SD). The significance level has been set for *p* value less than or equal to 0.05.

### 2.5. Reliability and Validity

The psychometric characteristics of the tool were assessed following the Consensus-Based Standards for the Selection of Health Status Measurement Instruments (COSMIN) checklist [44]. In order to assess test-retest reliability, a randomly chosen subgroup of the sample was evaluated twice. The time interval between test and retest should be sufficiently short in order to support the assumption that participants remain stable, yet sufficiently long to prevent participants from remembering the answers given in the first administration.

For the evaluation of the interrater reliability, two different professionals evaluated the same participant at the same time. To determine whether the test was reliable, the interclass correlation coefficient (ICC) was calculated. The ICC ranges from 0 (no agreement) to 1 (perfect agreement) and has been interpreted as follows: 0.00–0.25, little, if any, correlation; 0.26–0.49, a low correlation; 0.50–0.69, a moderate correlation; 0.70–0.89, a high correlation; and 0.9–1, a very high correlation.

Construct validity was evaluated examining the comparison with the gold standard SF-12 health survey, using Pearson's correlation coefficient to evaluate the correlation between the two tests. The Pearson's correlation coefficient was interpreted as follows: 0 indicated no linear relationship; +1/-1 indicated a perfect linear positive/negative relationship; a value between 0 and 0.3 (or 0 and -0.3) indicated a weak linear positive (negative) relationship through a shaky linear rule; values from 0.3 to 0.7 (-0.3 and -0.7) indicated a moderate positive (negative) linear relationship through a linear fuzzy-firm rule, and values between 0.7 and 1.0 (-0.7 and -1.0) indicated a strong positive (negative) linear relationship through a firm linear rule. Any *p* values less than or equal to 0.05 were considered statistically significant.

## 3. Results

### 3.1. Translation

After forward and backward translation and after the permission from the original author of the CIQ-R, the translated test was formed and approved. All items of the Italian version of the CIQ-R were either identical or similar in meaning to the original version's items (Appendix 1).

### 3.2. Participants

The Italian version of CIQ-R was administered to a total of 400 healthy individuals, recruited within the national region in the period ranging from June to August 2018. The sample included students from secondary schools and universities, but also professional workers, housewives, and pensioners.

All results and demographic characteristics are shown in Table 1.

### 3.3. Reliability and Validity

50 of the 400 included individuals were submitted to test-retest reliability procedures. The test-retest reliability was analyzed through Intraclass Correlation Coefficient (ICC), whose ranges are shown in Table 2. The excellent stability of the tool is also evident in the presence of minimal differences between total and partial average of the questionnaire at T0 and T1, as shown in the same table.

The internal consistency and the construct validity were calculated on all 400 included individuals. Cronbach's alpha coefficient of ≥0.70 was seen for the home integration subscale only, while for the total and the three subscales (social integration, productivity, ESN integration) Cronbach's alpha coefficients are <0.70. The results are shown in Table 3.

Pearson's test revealed a correlation between the total score of CIQ-R and Physical Component Summary (PCS) of the gold standard SF-12, between “social integration” subscale's score and PCS-12 and between “ESN integration” subscale's score and PCS-12, as shown in Table 4.

## 4. Discussion

The aims of this study are the translation, cultural adaptation, and validation of the Community Integration Questionnaire–Revised (CIQ-R) in Italian in a group of individuals with no clinical evidence of disability. The translation of the original CIQ-R was performed by applying internationally recognized methods [43].

This study provided the first CIQ-R's normative data in the Italian population showing that this is a reliable and valid instrument to measure “home, social, productivity integration, and electronic social networking” on people without serious and disabling pathologies.

About test-retest reliability, ICC has been calculated and its values are statistically significant, from ICC = 0.90 to ICC = 0.98: it means that the questionnaire has an excellent stability. This is reinforced also by the presence of minimal differences between total and partial average of the questionnaire at T0 and T1: it reveals that there was very little change over time in absolute scores. These results are also present in the questionnaire's validation study of its developer, where ICC values are from ICC = 0.70 to ICC = 0.84.

Cronbach's alpha coefficient shows a good internal consistency for the home integration (HI) subscale only (*α* = 0.82) so that we can consider it to be the most sensitive to measure community integration's level in healthy people. This result is very similar to those in other studies and validations, especially to those of the Croatian version of the CIQ-R, which was validated in healthy population and in TBI patients [23]. We can affirm that home integration subscale results to have the greatest sensitivity in measuring the level of individual's integration in family life and participation to housework's, such as shopping for groceries or other necessities, preparing meals, caring for the children, planning social arrangements (such as get-togethers with family and friends), or looking after personal finances (such as banking or paying bills).

The questionnaire has an excellent test-retest reliability: giving the same test to the same individuals on two occasions, its results are consistent over time. About construct validity, there is a correlation only with the Physical Component Summary (PCS12) of the SF-12, with Pearson's correlation coefficient (*r*) *p* > 0.05. More specifically, there is a correlation between PCS12 and the total score of the CIQ-R (*r* = 0.118 with *p* < 0.01), between PCS12 and SI subscale score (*r* = 0.121 with *p* < 0.01) and between PCS12 and ESN integration subscale score (*r* = 0.182 with *p* < 0.05). These results are similar with those of the Persian validation study [21]. The correlation of the CIQ-R with the PCS12 of the gold standard can be interpreted as that the level of reintegration of the individual is influenced by his/her level of functioning, physical health, and the presence or absence of any types of disease or pain.

Having these normative data for the healthy population, in future studies, it will be possible to compare these results with an administration on people with disabilities.

### 4.1. Limitations of the Study

This study has certain limitations; first, there is no correlation with a gold standard due to the fact that the specific tools for the community integration assessment are not validated in Italian yet. Moreover, CIQ-R data are limited to adults of working age (between 18 and 64): expansion of this range is required in order to expand the statistical survey.

## 5. Conclusion

The Italian version of the Community Integration Questionnaire–Revised (CIQ-R) has proven to be a reliable and valid assessment instrument to measure and value the global level of community integration in healthy population. Moreover, the CIQ-R is readily available; the time of its administration is short; it can be administered directly to the patient, either face-to-face or over the telephone; and it is sensitive to a wide variety of living situations.

The total results of this study showed statistically significant psychometric properties for use the Italian CIQ-R in clinical and research settings of healthy people.

## Figures and Tables

**Table 1:** Demographic characteristics of the 400 participants for the Italian version of the CIQ-R.

	Frequency (*N*)	%
Gender		
Female	253	63.3
Male	147	36.8
Age mean (±standard deviation)	37.18 ± 13.03	
Residence		
Metro	125	31.3
Regional/rural	275	68.8
Education		
University degree	154	38.5
Elementary school	65	16.3
High school	181	45.3
Annual household income		
≤30.000€	232	58.0
30.001€-60.000€	98	24.5
60.001€-90.000€	41	10.3
90.001€-120.000€	20	5.0
120.001€-150.000€	5	1.3
>150.000€	4	1.0
Living situation		
Lives with other people	358	89.5
Lives alone	42	10.5
Internet access		
Other types of Internet access	15	3.8
Home broadband connection	197	49.3
Internet-enabled smartphone or tablet	169	42.3
Metropolitan Wi-Fi	19	4.8

**Table 2:** Intraclass Correlation Coefficient (ICC) for the reliability of the Italian version of the CIQ-R.

	Test (T0) Mean (±SD)	Re-test (T1) Mean (±SD)	ICC	IC 95%
Home integration	6.1 (±2.99)	6.27 (±2.99)	0.981	(0.967-0.989)
Social integration	6.88 (±1.74)	6.88 (±1.94)	0.899	(0.821-0.943)
Productivity	5.02 (±1.49)	4.92 (±1.58)	0.928	(0.872-0.959)
Electronic social networking integration	3.69 (±1.55)	3.69 (±1.58)	0.956	(0.922-0.975)
Total	39.72 (±7.75)	39.90 (±7.25)	0.949	(0.91-0.971)

**Table 3:** Cronbach's *α* for internal consistency of the Italian version of the CIQ-R.

	Number of items	Cronbach's *α*	Mean (±standard deviation)
Home integration	6	0.82	5.88 (±2.90)
Social integration	5	0.395	6.72 (±1.87)
Productivity	2	0.003	4.89 (±1.60)
Electronic social networking integration	3	0.539	3.69 (±1.41)
Total	16	0.639	21.18 (±4.72)

**Table 4:** Pearson's correlation coefficient for the construct validity of the Italian version of the CIQ-R.

Pearson's correlation coefficient	PCS12	MCS12
Total	0.118^∗^	0.05
Home integration	0.028	0.036
Social integration	0.121∗	0.04
Productivity	0.083	0.033
Electronic social networking integration	0.184∗∗	0.056

PCS-12: Physical Component Summary (PCS) of the Short-Form-12 (SF-12) health survey; MCS-12: Mental Component Summary (MCS) of the SF-12 health survey. ^∗^*p* < 0.05. ^∗∗^*p* < 0.01.

## Data Availability

The data used to support the findings of this study are available from the corresponding author upon request.

## Additional Points

*Implication for Occupational Therapist's Clinical Practice*. Nowadays, the Community Integration Questionnaire–Revised (the CIQ-R) is one of the most commonly used outcome tools for measuring the participation and community integration of people with disabilities.

This study provides new information on the reliability and validity of this tool supporting its use among clinicians. They will be able to make informed decisions in setting up a therapeutic path for improving participation and community integration.

Normative data provided by this study on healthy population would not only allow the comparison of scores obtained by healthy individuals and people with disabilities but also the possibility to evaluate and set up an intervention on participation and community integration for people without disability.

This study on validation and crosscultural adaptation of the CIQ-R also provides researchers with a tool in an important and relevant area of study for future research and comparison between international studies.

## Supplementary Materials

Supplementary Materials Appendix 1 – Italian version of the Community Integration Questionnaire – Revised.
