# Supplementary material for: Crosscultural Validation of the Community Integration Questionnaire–Revised in an Italian Population
Source: Occup Ther Int. 2020 Aug 28;2020:8916541. doi: 10.1155/2020/8916541 (PMC7481919; doi:10.1155/2020/8916541)
Supplement: Supplementary Materials — Appendix 1 – Italian version of the Community Integration Questionnaire – Revised. [file 8916541.f1.docx]

Appendix 1 – Italian version of the Community Integration Questionnaire – Revised

*Prima di procedere alla compilazione del questionario, Le chiediamo di compilare la seguente tabella.*

| Nome |  |
| --- | --- |
| Cognome |  |
| Anni |  |
| Sesso | - M - F |
| Zona di residenza | - Città metropolitana - Paese |
| Educazione | - Licenza media - Licenza superiore - Diploma universitario |
| Reddito familiare annuo | - ≤ 30.000 € - 30.001 €- 60.000 € - 60.001 €- 90.000 € - 90.001 €- 120.000 € - 120.001 €- 150.000 € - > 150.000 € |
| Situazione abitativa | - Vivo da solo - Vivo con altri |
| Accesso ad Internet | - Connessione domestica a banda larga - Smartphone/tablet abilitati ad internet - Wi-Fi metropolitano - Altri tipi di connessioni |

1. Di solito, chi si occupa della spesa alimentare o dell’acquisto di altri prodotti in famiglia?

- Solo tu
- Tu e qualcun altro
- Qualcun altro

2. Di solito, chi prepara i pasti in famiglia?

- Solo tu
- Tu e qualcun altro
- Qualcun altro

3. In famiglia, chi solitamente svolge i lavori domestici?

- Li svolgi da solo
- Li svolgi insieme a qualcun altro
- Li svolge qualcun altro

4. Di solito, in casa chi si occupa dei bambini?

- Te ne occupi da solo
- Te ne occupi con qualcun altro
- Se ne occupa qualcun altro
- Non pertinente: in famiglia non ci sono ragazzi sotto i 17 anni

5. Di solito, chi organizza gli eventi sociali come le riunioni di famiglia o gli incontri con amici?

- Li organizzi da solo
- Li organizzi con qualcun altro
- Li organizza qualcun altro

6. Di solito, chi si occupa delle tue questioni econimiche come la banca o il pagamento delle bollette?

- Te ne occupi da solo
- Te ne occupi insieme a qualcun altro
- Se ne occupa qualcun altro

7. Approssimativamente, quante volte al mese ti dedichi allo shopping?

- 5 o più volte
- Da 1 a 4 volte
- Mai

8. Approssimativamente, quante volte al mese ti dedichi ad attività ricreative, come il cinema, lo

sport, andare al ristorante, etc?

- 5 o più volte
- Da 1 a 4 volte
- Mai

9. Approssimativamente, quante volte al mese solitamente fai visita ai tuoi amici o ai tuoi parenti?

- 5 o più volte
- Da 1 a 4 volte
- Mai

10. Quando ti dedichi ad attività ricreative, di solito lo fai da solo o in compagnia?

- Il più delle volte da solo
- Il più delle volte con la famiglia
- Il più delle volte con amici che hanno una disabilità
- Il più delle vlte con amici che non hanno una disabilità
- Con la famiglia e gli amici

11. Hai un caro amico/a con cui confidarti?

- Si
- No

12. Con quale frequenza esci?

- Quasi ogni giorno
- Quasi ogni settimana
- Raramente/mai (meno di una volta a settimana)

13. Per favore, spunta la risposta che meglio corrisponde alla tua attuale situazione di lavoro

(nell’ultimo mese):

- Lavoro a tempo pieno (più di 20 ore a settimana)
- Lavoro part-time (20 ore a settimana o meno)
- Disoccupato, ma sono attivamente impegnato nella ricerca di un lavoro
- Disoccupato, non sono impegnato nella ricerca di un lavoro
- Non pertinente, pensionato/a per età

14. Per favore, spunta la risposta che meglio corrisponde ai tuoi attuali impegni scolastici o alla tua

situazione formativo-professionale (nell'ultimo mese):

- A tempo pieno
- Part-time
- Non frequento la scuola né un corso/programma di formazione professionale
- Non pertinente, pensionato per età

15. Negli ultimi mesi, con quale frequenza sei stato impegnato in attività di volontariato?

- 5 volte o più
- Da 1 a 4 volte
- Mai

16. Nell’ultimo mese, con quale frequenza hai usato Internet per metterti in contatto con gli amici e

conoscenti (ad esempio: e-mail, social netwok come Facebook/Twitter etc)?

- Ogni giorno/molto frequentemente
- Quasi ogni settimana
- Raramente/Mai

17. Con quale frequenza usi internet per fare videochiamate (ad esempio: Skype, FaceTime etc)?

- Ogni giorno/molto frequentemente
- Quasi ogni settimana
- Raramente/Mai

18. Con quale frequenza contatti i tuoi amici o conoscenti mediante messaggi o chiamate con il tuo

telefono?

- Ogni giorno/molto frequentemente
- Quasi ogni settimana
- Raramente/Mai
